# Disparities in liver transplant waitlist characteristics and outcomes among Hispanic compared to non-Hispanic adults

**DOI:** 10.3389/frtra.2025.1592516

**Published:** 2025-08-20

**Authors:** Monica Tincopa, Jordan L. Pace, Fanny Delebecque, Kelly Torosian, Denya Arellano, Maria Elena Martinez, Irine Vodkin, Veeral Ajmera

**Affiliations:** ^1^ Department of Medicine, Division of Gastroenterology and Hepatology, MASLD Research Center, University of California San Diego, San Diego, CA, United States; ^2^ Department of Medicine, School of Medicine, California University of Science and Medicine, Colton, CA, United States; ^3^ Department of Medicine, University of California San Diego, San Diego, CA, United States; ^4^ Herbet Wertheim School of Public Health, University of California San Diego, San Diego, CA, United States

**Keywords:** health equity, Hispanic, mortality, cirrhosis, referral

## Abstract

**Background:**

Social determinants of health (SDOH) and transplant center characteristics have been associated with access to liver transplantation (LT) for Hispanic individuals. The aim of this study was to identify waitlist characteristics and correlates of odds of LT and waitlist removal by Hispanic ethnicity.

**Methods:**

This was a single-center cohort study of adults listed for LT between January 2018–December 2020. Demographic, clinical, and SDOH were analyzed using logistic regression.

**Results:**

375 patients were included. 52.5% (*N* = 197) were Hispanic. At time of listing, Hispanic patients had significantly higher BMI, prevalence of diabetes and metabolic dysfunction associated steatohepatitis. Rates of substance use were significantly lower and time of last drink to listing was significantly longer (641 vs. 391 days, *p* = 0.0007) in Hispanic adults. Rates of LT and waitlist removal did not significantly differ by Hispanic ethnicity (46.9% vs. 46.1% and 35% vs. 36.5%, respectively). Hepatocellular carcinoma (OR 3.28) was associated with odds of LT whereas employment status predicted waitlist removal.

**Conclusions:**

Distribution on the waitlist, LT and waitlist removal did not differ by Hispanic ethnicity. Hispanic patients had significantly longer time from last drink to listing, suggesting referral bias. Public health interventions to optimize LT referral are needed to increase health equity.

## Background

The Hispanic population is one of the fastest growing in the United States (US), accounting for approximately 20% of the population (1). Hispanic adults are disproportionately impacted by chronic liver disease, with the most pronounced disparities noted in prevalence of metabolic dysfunction–associated steatotic liver disease (MASLD) and its more aggressive subtype metabolic dysfunction–associated steatohepatits (MASH) (2). Despite this increased prevalence in liver disease with associated risk of need for liver transplantation (LT), prior studies have demonstrated lower rates of referral for LT, LT listing and rates of LT for Hispanic individuals (3–6). The mechanisms underlying these disparities in LT access are multifactorial and also involve intersectionality of several SDOH factors disproportionately impacting the Hispanic community in the US.

Access to healthcare is one SDOH that contributes strongly to disparities throughout the medical system, including LT. Patients with public forms of insurance have lower likelihood of referral for LT, and Hispanic individuals are less likely to have private insurance (7, 8). Both implicit and explicit biases and other forms of structural racism also impact referral and overall care management in chronic disease. From a liver transplant perspective, this manifests most strongly in forms of liver disease associated with health behaviors including alcohol-associated liver disease (ALD) (9, 10). Once referred and listed, several factors have been identified as impacting differences in rates of LT and waitlist removal for minority populations. Low socioeconomic status, public insurance or lack of insurance, and rural locality, factors that commonly impact Hispanic populations, have been independently associated with lower likelihood of LT and higher LT waitlist mortality (6, 11–14).

While current literature, including analyses of large databases such as the Scientific Registry of Transplant Recipients (SRTR), has shown racial and ethnic disparities in LT referral, listing, LT rates and waitlist outcomes, results specific to these outcomes for Hispanic individuals have been discordant across studies. For example, analysis of 24,595 LT from the National Inpatient Sample (NIS) found that Hispanic individuals had increased rates of LT compared to White patients [adjusted odds ratio (aOR) 1.16] (14). The specific factors identified as playing a causal role in disparities for Hispanic patients in need of LT have also been inconsistent in the literature. Therefore, the aim of this study was to identify factors impacting LT access including waitlist characteristics, correlates of odds of LT and waitlist removal by Hispanic ethnicity in a large, ethnically diverse academic transplant center database in which more detailed assessment of psychosocial and SDOH factors was available for analysis.

## Material and methods

### Study population and design

This retrospective cohort study included adults age 18 or older listed for LT between January 2018 and December 2020 at an academic transplant center in an ethnically diverse setting. Exclusion criteria included those listed as status 1A and patients who underwent prior LT. Our center utilizes a variety of grafts including donation after circulatory (DCD) and brain death (DBD) as well as living donors and extended criteria donors (ECDs) with graft acceptance evaluated by transplant surgery in consultation with transplant hepatology based on individual recipient characteristics. All data elements analyzed in the study were obtained through structured review of our EMR system. Demographic, clinical, psychosocial, and SDOH data were gathered through a retrospective review of electronic medical records. Data extracted from chart review consisted of past medical history, family history, social history, multidisciplinary pre-transplant workup, post-transplant follow-up notes, imaging, laboratory and other diagnostic testing results and information regarding removal from waitlist, death, or LT. Cardiac parameters, including ejection fraction and right ventricular systolic pressure (RVSP), were primarily obtained from transthoracic echocardiogram (TTE) performed as part of routine pre-transplant cardiovascular evaluation to reflect potential cardiopulmonary contributors to listing and transplantation decisions. Heart catheterization data was conducted in select cases when indicated based on initial screening. Psychiatric and substance use history included history of failed rehabilitation, time (in days) between last drink and listed for transplant, Alcohol Use Disorders Identification Test (AUDIT) score, history of mental health conditions, marijuana use, other substance use, tobacco use, and Stanford Integrated Psychosocial Assessment Tool (SIPAT). Karnofsky scores, used to assess functional status, were collected as well. SDOH included annual household income, education level, current employment status, marital status, and whether the patient had private insurance. Annual income was categorized as <$25,000, $25–50,000, $50–100,000, and >$100,000. Education level was classified as less than high school, completed high school, some college or associates degree, college graduate, or advanced degree. Current employment status included employed vs. unemployed. Marital status was defined as either married/in a long-term partnership or not. Ethics approval for this study was provided by our Institutional Review Board.

### Statistical analysis

Chi-squared analysis was used to compare demographic, clinical, psychosocial, and SDOH between Hispanic and non-Hispanic candidates at time of listing. Univariate and multivariate logistic regression analyses were conducted to examine characteristics associated with odds of LT and waitlist removal. Statistical analysis was performed using STATA software, with statistical significance defined as *p* < 0.05.

## Results

### Patient characteristics at time of LT listing

A total of 375 patients listed for LT were included in this study, of whom 52.5% (*N* = 197) were Hispanic. The non-Hispanic cohort (*N* = 178) consisted of 76.4% (*N* = 136) White, 4.5% (*N* = 8) Black, 9.5% (*N* = 17) Asian, and 9.5% (*N* = 17) other race (including American Indian, Alaska Native, Native Hawaiian or other Pacific Islander and mixed race not including Hispanic). The cohort consisted of 224 males (59.7%) with a median age of 57 [interquartile range (IQR) 50–63], median body mass index (BMI) of 27.5 with 24% having type II diabetes (Table 1). The most common etiologies of liver disease were ALD (41.2%), hepatitis C virus (HCV) 20.8% and MASH (18.7%). Median model of end stage liver disease (MELD) Na at time of listing was 16 (IQR 10–24). At time of listing, Hispanic patients had higher BMI (28.2 vs. 26.2 kg/m^2^, *p* = 0.009), percentage of diabetes (42.6% vs. 24.1%, *p* = <0.001), and MASH as indication for LT (26.9% vs. 9.6%, *p* = <0.001). There were no statistically significant differences in MELDNa at listing, hepatocellular carcinoma (HCC), forms of decompensation or functional status as assessed by Karnofsky score between Hispanic and non-Hispanic candidates. From a cardiac perspective, Hispanic patients had significantly lower right ventricular systolic pressure.

**Table 1:** Baseline characteristics of patients listed for liver transplant.

Variable (median, IQR)	Overall (*N* = 375)	Hispanic (*N* = 197)	Non-Hispanic (*N* = 178)	*P* value
Demographics and medical co-morbidities				
Age (yr)	57 (50–63)	57 (51–63)	58 (49–64)	0.52
Male sex	224 (59.7%)	114 (57.8%)	110 (61.8%)	0.34
BMI	27.5 (23.9–32.5)	28.2 (24.5–33.6)	26.6 (23.1–32.2)	**0**.**009**
Diabetes	127 (33.8%)	84 (42.6%)	43 (24.1%)	**<0**.**001**
Liver disease history				
Etiology of liver disease				**<0**.**001**
ALD	154 (41.2%)	66 (33.5%)	88 (49.7%)	
MASH	70 (18.7%)	53 (26.9%)	17 (9.6%)	
HCV	78 (20.8%)	44 (22.3%)	34 (19.2%)	
HCV + ALD	26 (6.9%)	14 (7.1%)	12 (6.7%)	
HBV	8 (2.1%)	2 (1%)	6 (3.3%)	
Cholestatic/AIH	19 (5.1%)	8 (4.1%)	11 (6.2%)	
Other	19 (5.1%)	10 (5.1%)	9 (5.1%)	
Decompensation				
Ascites	260 (70.2%)	132 (68.4%)	128 (72.3%)	0.41
h/o variceal bleed	145 (39.5%)	84 (43.7%)	61 (34.8%)	0.08
HE	232 (62.2%)	126 (64.2%)	106 (59.9%)	0.32
HCC	98 (26.7%)	57 (29.8%)	41 (23.4%)	0.16
Karnofsky	70 (70–80)	70 (70–90)	70 (60–80)	0.14
MELDNa at listing	16 (10–24)	16 (11–23)	16 (11–23)	0.49
Cr	0.91 (0.7–1.39)	0.87 (0.67–1.41)	0.94 (0.74–1.35)	0.07
HCC features at listing				
Largest tumor	2.6 (2.1–3.6)	2.6 (2.1–3.6)	2.5 (2–3.6)	0.44
Number of tumors	1 (1–2)	1 (1–2)	1 (1–2)	0.48
Transplant testing				
LHC	52 (14.1%)	25 (12.8%)	27 (15.5%)	0.44
EF	67 (64–72%)	67 (64–73)	67 (64–71.5)	0.56
RVSP	29 (25–35)	28 (24–33)	30 (25–37)	**0**.**03**
Mental health and substance use history				
For ALD				
h/o failed rehab	65 (35.1%)	29 (34.5%)	36 (35.6%)	0.87
Time last drink to list	500 (271–1,227)	641 (330–2,530)	391 (229–810)	**0**.**0007**
AUDIT score	0 (0–4.5)	0 (0–2)	0 (0–13)	**0**.**008**
Mental health condition	84 (21.2%)	38 (18%)	46 (24.8%)	0.09
History of substance abuse				
Marijuana	128 (34.1%)	48 (24.3%)	80 (44.9%)	**<0**.**001**
Other illicit substances	107 (28.5%)	51 (25.9%)	56 (31.5%)	0.23
History of tobacco use	183 (48.8%)	92 (46.7%)	91 (51.1%)	0.39
SIPAT	28 (20–38)	27 (20–36)	29 (20–400	0.27
SDOH at Listing				
Level of formal education				**<0**.**001**
<High school	69 (17.2%)	63 (29.4%)	6 (3.2%)	
High school	148 (37%)	90 (42.1%)	58 (31.2%)	
Some college/associate	109 (27.2%)	53 (24.3%)	57 (30.6%)	
College graduate	47 (11.7%)	6 (2.8%)	41 (22%)	
Advanced degree	21 (11.3%)	2 (1%)	21 (11.3%)	
Currently employed	98 (24.7%)	38 (17.9%)	60 (32.6%)	**0**.**001**
Married/long-term partner	245 (61.7%)	136 (63.5%)	109 (59.6%)	0.41
Annual income (*n* = 137)				**0**.**01**
<25K	105 (75.5%%)	75 (78.1%)	30 (69.7%)	
25–50K	17 (12.2%)	13 (13.5%)	4 (9.3%)	
50–100,000	13 (9.3%)	8 (8.3%)	5 (11.6%)	
>100,000	4 (2.8%)	0	4 (9.3%)	
Private insurance	142 (35.5%)	56 (26.1%)	86 (46.2%)	**<0**.**001**

Bold values indicate statistical significance (*p* < 0.05). BMI, body mass index; ALD, alcohol associated liver disease; MASH, metabolic dysfunction-associated steatohepatitis; HCV, hepatitis C virus; HBV, hepatitis B virus; AIH, autoimmune hepatitis; HE, hepatic encephalopathy; HCC, hepatocellular carcinoma; MELD, model for end stage liver disease; Cr, creatinine; LHC, left heart catheterization; EF, ejection fraction; RVSP, right ventricular systolic pressure; AUDIT, alcohol use disorders identification test; SIPAT, Stanford Integrated Psychological Assessment.

### Time of LT listing psychosocial factors and SDOH

Prevalence of mental health conditions were similar between the two groups. From a substance use perspective, Hispanic patients had significantly lower rates of marijuana use (24.3% vs. 44.9%, *p* = <0.001) and similar rates of use of tobacco and other illicit substances compared to non-Hispanic individuals. SIPAT scores were similar between Hispanic and non-Hispanic candidates. Among patients listed for ALD, Hispanic patients had significantly lower Alcohol Use Disorder Identification Test (AUDIT) scores and had significantly longer duration from time to last drink to listing (641 days vs. 391 days, *p* = 0.007). SDOH substantially differed by Hispanic ethnicity with Hispanic patients having fewer years of formal education (*p* = <0.001), rates of employment (17.9% vs. 32.6%, *p* = 0.001), annual household income (*p* = 0.01), and private insurance (26.1% vs. 46.2%), *p* < 0.001).

### Transplantation and waitlist removal

**Figure 1:**
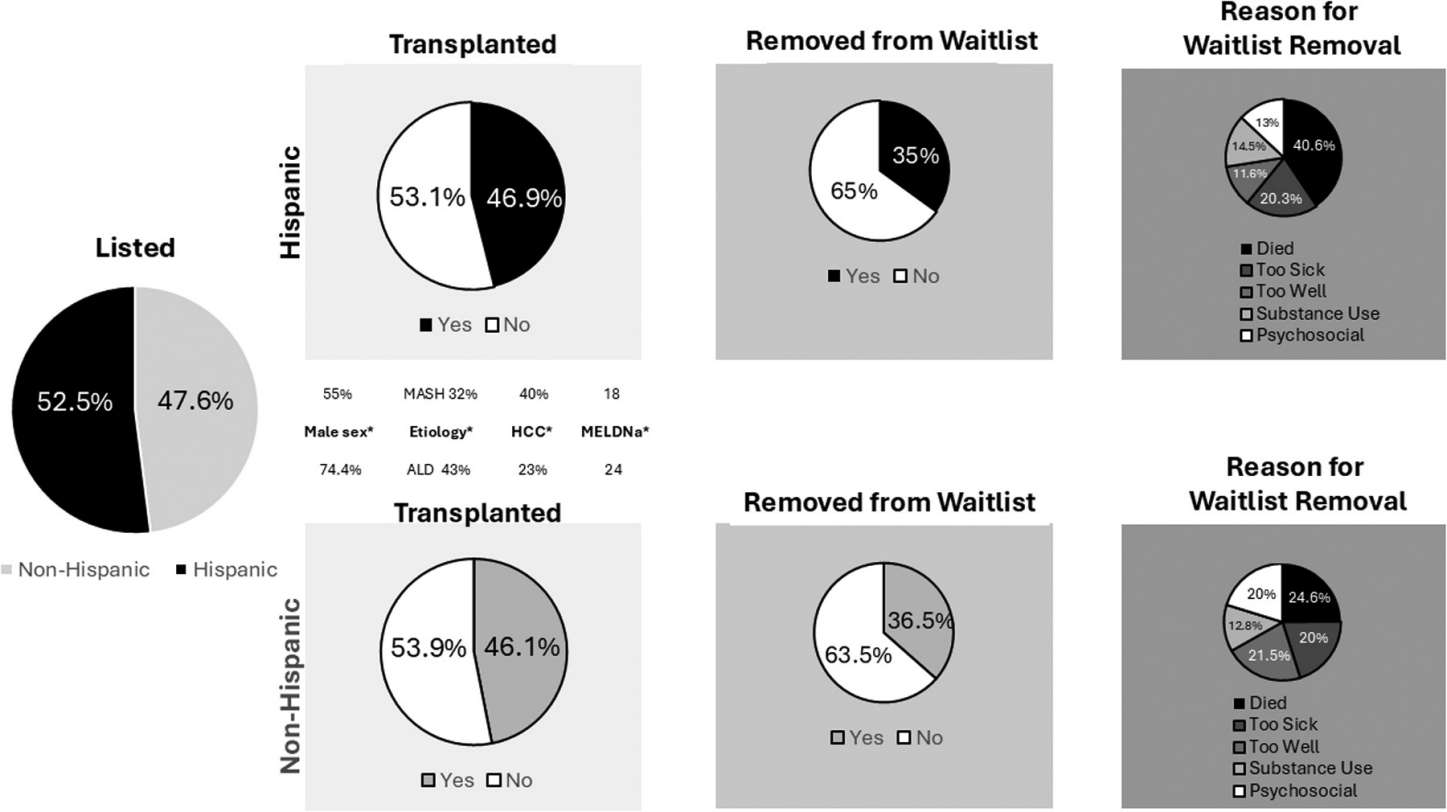
Listing, transplantation and removal from LT waitlist by Hispanic ethnicity. *denotes statistically significant difference in variable between groups.

Rates of transplantation did not significantly differ by Hispanic ethnicity (46.9% vs. 46.1%, *p* = 0.98) (Figure 1). Similarly, time from listing to transplant did not vary based on Hispanic ethnicity (214 days in Hispanic vs. 184.5 days in non-Hispanic candidates, *p* = 0.12). No significant differences in multiorgan transplantation, use of DCD or high-risk donor organs were noted between Hispanic and non-Hispanic candidates. The majority of grafts were DBD donors. Two living donor grafts were performed in this cohort. LT recipients in the Hispanic cohort were less often male (54.9% vs. 74.4%, *p* = 0.008), more commonly transplanted for MASH, and had lower biologic MELDNa at time of LT (18 vs. 24, *p* = 0.003) in the setting of higher HCC indications (40% vs. 23.2%, *p* = 0.003). Removal from the waitlist also did not significantly differ by Hispanic ethnicity (35% vs. 36.5%, *p* = 0.87). Indications for removal from the waitlist did not statistically significantly differ, though 60.9% of Hispanic patients were removed due to death or being too sick compared to 44.6% of non-Hispanic patients (*p* = 0.04, Figure 1).

On univariate logistic regression, several characteristics were significantly associated with odds of transplantation (Table 2). The presence of HCC [odds ratio (OR) 1.65, 95% confidence interval (CI) 1.03–2.63], MELDNa at listing (OR 1.04, 95% CI 1.02–1.06), and AB blood type (OR 4.02, 95% CI 1.05–15.3) were all associated with higher odds of LT. Of note, Karnofsky scores were also associated with odds of transplant (OR 0.98, 95% CI 0.97–0.99). The only psychosocial factor associated with odds of LT was time from last drink to listing (OR 0.99, 95% CI 0.99–0.99). For odds of removal from the waitlist, history of ascites (OR 1.68, 95% CI 1.01–2.81), time from last drink to listing (OR 1.01, 95% CI 1.01–1.01) and current employment status (OR 0.53, 95% CI 0.30–0.93) were the only significant factors. On multivariate logistic regression, HCC (OR 10.2, 95% CI 1.15–90.44) was associated with increased odds of transplant (Table 3). The only factor independently associated with odds of removal from the waitlist on multivariate analysis when accounting for baseline differences in the Hispanic vs. non-Hispanic cohorts was employment status.

**Table 2:** Correlates of transplant or removal from waitlist^a^ on logistic regression.

Variable (median, IQR)	Odds ratio (95% CI)	*p*-value	Odds ratio (95% CI)	*p*-value
	Transplanted		Removed from waitlist	
Age (yr)	1.00 (0.98–1.02)	0.47	1.00 (0.98–1.02)	0.54
Male sex	1.41 (0.92–2.13)	0.10	0.81 (0.52–1.27)	0.37
Hispanic ethnicity	0.99 (0.66–1.49)	0.98	0.89 (0.57–1.39)	0.62
BMI	1.01 (0.97–1.04)	0.49	0.97 (0.93–1.00)	0.14
Diabetes	1.29 (0.84–1.98)	0.23	0.84 (0.52–1.35)	0.48
Etiology of liver disease				
ALD (1) (reference)				
MASH (2)	1.66 (0.94–2.93)	0.08	0.79 (0.42–1.49)	0.48
HCV (3)	1.56 (0.90–2.70)	0.11	0.78 (0.43–1.44)	0.44
HCV + ETOH (4)	1.27 (0.55–2.93)	0.57	1.57 (0.67–3.67)	0.29
HBV (5)	0.49 (0.09–2.53)	0.39	2.14 (0.51–8.92)	0.29
Cholestatic/AIH (6)	1.07 (0.41–2.83)	0.87	0.76 (0.26–2.24)	0.62
Other (7)	2.04 (0.77–5.36)	0.14	0.40 (0.11–1.44)	0.16
Decompensation				
Ascites	0.72 (0.46–1.13)	0.16	1.68 (1.01–2.81)	**0**.**04**
h/o GIB	1.45 (0.95–2.22)	0.07	1.02 (0.65–1.62)	0.90
HE	0.98 (0.64–1.49)	0.93	1.50 (0.94–2.40)	0.08
HCC	1.65 (1.03–2.63)	**0**.**03**	1.28 (0.78–2.10)	0.32
Karnofsky	0.98 (0.97–0.99)	**0**.**001**	1.00 (0.99–1.01)	0.51
MELDNa at listing	1.04 (1.02–1.06)	**<0**.**001**	1.01 (0.98–1.03)	0.32
Blood type				
A (reference)				
B	1.75 (0.89–3.41)	0.10	0.93 (0.43–2.00)	0.86
O	0.84 (0.53–1.32)	0.45	1.55 (0.94–2.56)	0.08
AB	4.02 (1.05–15.30)	**0**.**04**	0.52 (0.11–2.48)	0.41
Psychiatric disease	1.18 (0.72–1.92)	0.49	1.09 (0.64–1.85)	0.74
History of substance abuse	0.69 (0.45–1.04)	0.07	1.15 (0.74–1.80)	0.52
Time last drink to list	0.99 (0.99–0.99)	**0**.**02**	1.01 (1.01–1.01)	**0**.**04**
History of failed rehab	1.12 (0.59–2.10)	0.71	1.06 (0.55–2.04)	0.85
SIPAT	0.99 (0.98–1.01)	0.73	1.01 (0.99–1.03)	0.09
Level of formal education				
<high school (reference)				
High school	1.08 (0.59–1.99)	0.78	1.36 (0.70–2.64)	0.35
Some college/associate	1.11 (0.59–2.09)	0.74	1.07 (0.53–2.16)	0.84
College graduate	1.70 (0.78–3.71)	0.18	0.88 (0.36–2.13)	0.78
Advanced degree	0.99 (0.37–2.61)	0.99	0.75 (0.23–2.29)	0.59
Currently employed	1.06 (0.66–1.70)	0.79	0.53 (0.30–0.93)	**0**.**02**
Married/long-term partner	1.23 (0.80–1.88)	0.32	0.79 (0.50–1.24)	0.31
Annual income (*n* = 131)				
<25 K (reference)				
25–50 K	1.65 (0.58–4.69)	0.34	0.65 (0.19–2.17)	0.48
50–100,000	1.84 (0.56–6.05)	0.31	0.38 (0.08–1.85)	0.23
>100,000	0.38 (0.03–3.83)	0.41	0.70 (0.07–7.1)	0.77
Private insurance	0.83 (0.55–1.27)	0.41	1.56 (0.97–2.51)	0.06

Bold values indicate statistical significance (*p* < 0.05). BMI, body mass index; ALD, alcohol associated liver disease; MASH, metabolic dysfunction-associated steatohepatitis; HCV, hepatitis C virus; HBV, hepatitis B virus; AIH, autoimmune hepatitis; HE, hepatic encephalopathy; HCC, hepatocellular carcinoma; MELD, model for end stage liver disease; Cr, creatinine; LHC, left heart catheterization; EF, ejection fraction; RVSP, right ventricular systolic pressure; AUDIT, alcohol use disorders identification test; SIPAT, Stanford Integrated Psychological Assessment.

^a^ Includes death, medical worsening, substance relapse and psychosocial factors.

**Table 3:** Multivariate analysis of correlates of transplant or removal from waitlist^a^ including SDOH differences in table 1 by Hispanic ethnicity.

Variable (median, IQR)	Odds ratio (95% CI)	*p*-value	Odds ratio (95% CI)	*p*-value
	Transplanted		Removed from waitlist	
Age (yr)	0.97 (0.87–1.08)	0.66	1.04 (0.95–1.14)	0.30
Male sex	1.68 (0.15–18.5)	0.67	0.47 (0.08–2.74)	0.40
Hispanic ethnicity	4.62 (0.42–50.02)	0.20	1.41 (0.87–2.27)	0.15
HCC	10.20 (1.15–90.44)	**0**.**03**		
Karnofsky	0.93 (0.86–1.01)	0.08		
MELDNa at listing	1.07 (0.96–1.18)	0.17		
Ascites			3.18 (0.23–42.78)	0.38
Blood Type				
A (reference)				
B	12.89 (0.26–635.14)	0.19		
O	7.64 (0.74–77.99)	0.08		
Time last drink to list	1.00 (0.99–1.00)	0.59	1.00 (0.99–1.00)	0.67
High school or less formal				
Education	3.05 (0.42–22.06)	0.26	0.35 (0.08–1.47)	0.15
Currently employed	1.08 (0.82–14.24))	0.96		**Predicts perfectly**
Household income				
<$50,000	1.30 (0.70–24.39)	0.85	2.95 (0.19–6.84)	0.43
Private insurance	0.11 (0.01–1.27)	0.07	0.93 (0.12–6.87)	0.95

Bold values indicate statistical significance (*p* < 0.05). HCC, hepatocellular carcinoma; MELD, model for end stage liver disease.

^a^ Includes death, medical worsening, substance relapse and psychosocial factors.

## Discussion

### Main findings

Analysis of patients listed for LT at a large, academic transplant center with a high density of Hispanic/Latino patients in its referral area demonstrated no significant differences in rates of LT and removal from the LT waitlist for Hispanic compared to non-Hispanic patients. This was despite the Hispanic cohort having statistically significantly higher rates of medical co-morbidities (higher BMI, type II diabetes) and SDOH factors that have been associated with lower access to LT (fewer years of formal education, lower annual income, lower rates of employment and less private insurance). The finding of equitable distribution on the LT waitlist, rates of LT and removal from the waitlist despite these potentially challenging medical and SDOH factors may in part be explained by the protective psychosocial factors noted in the Hispanic group. Further, these comparable findings between Hispanic and non-Hispanic patients despite identified SDOH found among the Hispanic cohort could possibly be explained by the strategic structure of our health system designed to mitigate barriers to access. Studies have shown that the effects of SDOH can vary by region, reflecting differences in local healthcare delivery models, referral patterns, and transplant center practices (15, 16). The single center used for this study has established community outreach programs as well as culturally tailored services, which may explain the apparent attenuation of disparities generally seen in broader national datasets. These services include the use of bilingual navigators, interpreter services, and expedited virtual consult pathways that support earlier and more equitable access to evaluation. Our findings highlight the importance of examining healthcare delivery models that may reduce the effects of SDOH and identifying which specific interventions within this health network have been most impactful, potentially serving as models for broader implementation.

Important differences in referral patterns were noted impacting access to LT, specifically significantly longer duration of time from last drink to listing for Hispanic candidates, highlighting potential biases and SDOH factors contributing to delay in referral for this population or lack of up to date knowledge regarding LT protocols for ALD for referring providers. On multivariate analysis, only HCC predicted odds of transplant whereas only SDOH factors, employment status, predicted odds of removal from the LT waitlist.

### In context with current literature

Our findings build on the existing literature by addressing discordant findings regarding distribution on the LT waitlist, rates of LT and removal from the LT waitlist between Hispanic and non-Hispanic LT candidates. Discrepancies in results, particularly of analyses from larger nationwide databases, may reflect lack of more detailed assessments of psychosocial and SDOH factors impacting health equity. Differences in results across studies may also be impacted by comparator groups used with some studies comparing only to non-Hispanic White participants and others having non-Hispanic of any other race/ethnicity as the comparator. In this study, the non-Hispanic cohort consisted of 76% White individuals, with the remaining including Black. Asian and other race/ethnicity. We opted to keep participants from other non-Hispanic ethnicities in the comparator group to enhance power and to ascertain the impact of variables on outcomes of interest by Hispanic ethnicity alone. Our analyses outlined relevant factors that may impact LT candidacy that ultimately were not reflected in significant differences in SIPAT scores. Potential biases in psychosocial assessments and global LT listing patterns were shown in a recent study that found that Hispanic candidates were more likely to be denied listing due to psychosocial concerns compared to non-Hispanic white patients (10).

### Implications for clinical care and research

Given the rising burden of MASLD, particularly among Hispanic populations, the implications of our findings warrant further consideration. While this study was not designed to stratify outcomes by liver disease etiology, prior work by our group demonstrated that patients with MASH had similar rates of liver transplantation and waitlist removal compared to other etiologies, but experienced higher waitlist mortality (17). The higher prevalence of MASLD among Hispanic patients may contribute to observed disparities in access and outcomes. These findings underscore the importance of addressing MASLD-related social determinants of health and ensuring equitable transplant evaluation.

Once contributors to health disparities across groups are identified, it is critical to identify pragmatic, actionable interventions to address these factors to improve health equity. Several LT centers have designed programs focused on LT for Hispanic individuals with goals of increasing referral, listing, LT rates and improving long-term clinical outcomes. One such program in Texas resulted in increases in referral for LT, though the proportion of Hispanic patients undergoing LT dropped due to financial barriers (18). Our center does not have any restrictions on accepting public insurance, including Medicaid, in an effort to obviate potential disparities seen in regards to access to transplant and waitlist maintenance. Additionally, our center has implemented a systemwide initiative designed to identify barriers to access and to improve equity. Barriers to access were found to be multifactorial, including both patient-level factors and structural factors within the healthcare system including delays in referral triage, language barriers, inconsistent navigation of the healthcare system, and limited appointment flexibility. As a result, our center has implemented several interventions including virtual expedited consult clinics, self-scheduling portals, expanded interpreter services, and provider-to-provider e-consults that allow primary care physicians to initiate specialty evaluation without requiring in-person visits. These system-level changes have led to measurable improvements in specialty care access at our center and highlight the importance of addressing healthcare delivery, in addition to addressing patient-level factors. Current literature indicates that while some centers have implemented similar initiatives, there is limited research evaluating their effectiveness in significantly increasing transplantation rates among the Hispanic population.

Therefore, these models highlight the potential for transplant centers to identify and systematically address barriers to care and the need to evaluate the effectiveness of such interventions.

From an outcomes viewpoint, these disparities have significant implications as prior studies have shown better post-LT patient and graft survival in Hispanics compared to non-Hispanic LT recipients (5, 19). Similarly, a lower rate of biochemical alcohol relapse has been documented in Hispanic patients compared to White patients, further suggesting disparity in the referral and selection process (20). Future studies are needed to determine whether these better outcomes are secondary to unidentified protective factors in this patient population that may have implications on transplant selection. From a public health viewpoint, it is necessary to identify interventions to not only increase referrals for LT but mechanisms targeted at minimizing SDOH factors impeding LT listing and maintenance on the waitlist.

### Strengths and limitations

A main strength of this study is the detailed assessment of both potential risk and beneficial factors impacting LT and waitlist removal. Specifically, we were able to abstract relevant psychosocial and SDOH factors that are frequently excluded or minimally captured in larger databases. Through this methodology we were able to highlight key differences in referral patterns for patients with ALD in Hispanic compared to non-Hispanic patients. An inherent limitation of any retrospective study results from potential missingness in data capture as a result of differential level of detail documented for each patient that was available for abstraction. The overall missingness for each variable of interest was minimal however given the comprehensive chart review performed by experienced research staff who are practitioners in liver transplant. Our transplant program has data from time of referral regarding listing and transplantation status. This was also a single center study from a diverse, large academic transplant center, and thus our findings may not be reflective of other transplant centers with more homogeneous patient populations. Given the high density of Hispanic individuals in our referral area, our center provided an ideal setting to investigate the outcomes of interest for this patient population. Our sample size was modest however, and this may impact the statistical power to detect differences across groups.

## Conclusions

In conclusion, in a large, ethnically diverse academic liver transplant center, rates of LT and waitlist removal did not differ by Hispanic ethnicity. Hispanic patients did appear to have delays in referral for ALD compared to non-Hispanic counterparts. Both risk and protective factors were identified in the Hispanic cohort that impact odds of LT. SDOH, particularly employment status, predicted removal from the waitlist, and was more common among Hispanic candidates. Given the increasing burden of MASLD, especially among Hispanic populations, and its strong association with socioeconomic and lifestyle factors, these findings highlight the importance of addressing SDOH that impact transplant access. Tailored, culturally sensitive programs aimed at increasing referral and listing for LT have shown promise for Hispanic patients, though downstream benefits of increased rates of transplantation have thus far remained works in progress. Designing programs that both mitigate risk factors and enhance the benefits of protective characteristics may advance progress towards health equity for Hispanic patients in need of LT.

## Data Availability

The raw data supporting the conclusions of this article will be made available by the authors, without undue reservation.
