# Correction: Disparities in liver transplant waitlist characteristics and outcomes among Hispanic compared to non-Hispanic adults

**DOI:** 10.3389/frtra.2025.1720964

**Published:** 2025-10-21

**Authors:** Monica Tincopa, Jordan L. Pace, Fanny Delebecque, Kelly Torosian, Denya Arellano, Maria Elena Martinez, Irine Vodkin, Veeral Ajmera

**Affiliations:** ^1^ Department of Medicine, Division of Gastroenterology and Hepatology, MASLD Research Center, University of California San Diego, San Diego, CA, United States; ^2^ Department of Medicine, School of Medicine, California University of Science and Medicine, Colton, CA, United States; ^3^ Department of Medicine, University of California San Diego, San Diego, CA, United States; ^4^ Herbet Wertheim School of Public Health, University of California San Diego, San Diego, CA, United States

**Keywords:** health equity, Hispanic, mortality, cirrhosis, referral

**Incorrect funding**

Funder name incorrect

An incorrect Funding statement was provided. The correct funder is outlined below the correct funding statement reads:

The author(s) declare that financial support was received for the research and/or publication of this article. MT is supported by the National Cancer Institute of the National Institutes of Health (U54CA285117 and U54CA2851) and ACTRI voucher funding. VA is supported by NIDDK (K23DK119460) and ACTRI voucher funding (UL1TR001442).

